# Perceptions of FDA-authorized e-cigarettes and use interest among young adults who do not use tobacco

**DOI:** 10.18332/tpc/190582

**Published:** 2024-07-29

**Authors:** Kathryn La Capria, Kristen R. Hamilton-Moseley, Lilianna Phan, Bambi Jewett, Kiana Hacker, Kelvin Choi, Julia Chen-Sankey

**Affiliations:** 1 Rutgers Institute for Nicotine and Tobacco Studies, New Brunswick, New Jersey, United States; 2 National Institute of Minority Health and Health Disparities, Bethesda, Maryland, United States; 3 Drexel University, Dornsife School of Public Health and College of Nursing and Health Professions, Philadelphia, Pennsylvania, United States; 4 Rutgers School of Public Health, Piscataway, New Jersey, United States

**Keywords:** e-cigarettes, tobacco, FDA authorization, FDA-authorized e-cigarettes, in-depth interview, young adults

## Abstract

**INTRODUCTION:**

It is unclear how young adults who do not use tobacco perceive FDA-authorized e-cigarettes for market entry. This study explored the perceptions and use interests of FDA-authorized e-cigarettes among this population to inform policy decision-making.

**METHODS:**

We conducted in-depth interviews with young adults in the US, aged 18–29 years, who do not use tobacco (n=25). Participants viewed images of FDA-authorized e-cigarettes and discussed their perceptions and interest in using these products. We used reflexive thematic analysis to analyze interview data.

**RESULTS:**

Many participants stated that they were not interested in using FDA-authorized e-cigarettes because they had little interest in using e-cigarettes in general. Additionally, almost all participants found the tobacco flavor and product design of these products unappealing, which further added to their disinterest. Most reported high trust in the FDA and its authorization process for e-cigarette market entry and considered FDA-authorized e-cigarettes safe to use. Most considered FDA-authorized products less harmful than other (unauthorized) e-cigarettes they saw in daily life but were not more interested in using the authorized products. When given the hypothetical scenario where FDA-authorized products come with a variety of fruit and candy flavors, most participants still expressed little interest in using them, mainly due to the high perceived harm from using any e-cigarette products.

**CONCLUSIONS:**

Although non-tobacco-using young adults in our study reported low interest in using FDA-authorized e-cigarettes for market entry and e-cigarettes in general, the FDA is recommended to continue to evaluate the impact of flavorings and packages on e-cigarette product appeal to reduce e-cigarette use among young people. The FDA should also examine strategies to effectively communicate the purpose of FDA authorization to the public and emphasize that it does not mean these products are ‘approved’ or safer than unauthorized products.

## INTRODUCTION

The prevalence of e-cigarette use among US young adults has increased in recent years. E-cigarette use prevalence in the past 30 days among this population was 13.7% in 2017 and soared to 23.7% in 2022^1^. This high prevalence warrants attention given that over half of young adults who reported e-cigarette use in recent years were those who had never smoked cigarettes^1^. Initiating e-cigarette use in young adulthood increases the risk of nicotine addiction, respiratory issues, and harm to the developing brain from nicotine exposure^2^. Additionally, e-cigarette initiation may lead to a more harmful form of tobacco use, including smoking cigarettes^3^. Therefore, young adults who do not use tobacco are a priority population for e-cigarette use prevention. It is important to explore how perceptions of FDA-authorized e-cigarettes for market entry (thereafter, FDA-authorized e-cigarettes), in particular, are perceived by young adults who do not use tobacco and how these authorizations impact e-cigarette use interest.

In 2021, the U.S. Food and Drug Administration (FDA) began authorizing new e-cigarettes for market entry with the purpose of promoting product switching among adults who smoke cigarettes^4^. E-cigarette companies can receive product authorization for market entry through a pathway called Premarket Tobacco Product Applications (PMTA)^5^. These applications present data that show whether the proposed e-cigarette product is suitable with respect to the protection of public health. When reviewing the applications, the FDA evaluates the potential risks and benefits of e-cigarette authorization for both those who currently use tobacco and those who do not use tobacco. Specifically, the FDA considers the likelihood that those who use tobacco will switch from their current product to the e-cigarette newly authorized for market entry and the likelihood that those who do not use tobacco will start using the authorized e-cigarette product^6^. Flavors, product designs, and marketing plans are also evaluated for those applications to understand if young people who do not use tobacco would be more or less likely to begin using the products^7^. Once an e-cigarette company receives FDA authorization for market entry, the e-cigarette can be marketed and sold in the country, and the FDA will continue to monitor the marketing and sales of the authorized product to see whether they are demonstrating protection to public health^5^. Since 2021, the FDA has authorized more than 20 e-cigarette products and devices from several brands (Vuse, NJOY, and Logic)^8^ for market entry and denied authorization to more than 1 million products^9^. Meanwhile, about 6.7 million new PMTAs were accepted for review as of September 2023^9^.

Despite having a rigorous review process in place, there is limited research regarding whether FDA authorization for market entry may mislead young adults who do not use tobacco to believe that FDA-authorized e-cigarettes are safe to use. This misunderstanding may lead to product experimentation and use, which can increase health risks related to nicotine exposure in young adulthood^2^. This assessment is crucial because FDA authorization does not imply that the products are deemed safe for use; it only means they are legally permitted to be marketed and sold in the US. Additionally, some researchers raised questions about the FDA’s justification that the benefits of these authorized e-cigarettes for those who smoke outweigh the risk of product initiation among those who do not use tobacco, including young people, asserting that the FDA’s analysis and decision-making for PMTAs should be re-evaluated^10,11^.

Few studies have assessed awareness, beliefs, and product perceptions surrounding e-cigarette authorization for market entry since the initiation of the PMTA review^12-14^. Results from two previous studies demonstrate that few participants were aware that e-cigarettes are regulated by the FDA^12,14^. Additionally, seeing messages or ads for FDA-authorized e-cigarettes was associated with the perception that FDA authorization means product approval or safety among adults who smoke and youth^12,14^. In contrast, findings from another study, which assessed perceptions of Vuse e-cigarette authorization on Twitter, convey that the general public has more negative than positive views of FDA e-cigarette authorization due to wariness of potential health risks^13^. In addition to limited research on this topic in general and varying results, there is a need for an in-depth qualitative exploration of FDA-authorized e-cigarette product perceptions and use interest among young adults who do not use tobacco.

To address these research gaps, we conducted one-on-one, in-depth interviews with young adults who do not use tobacco to explore the perceived appeal and use interests of FDA-authorized e-cigarette products. The results from this study can be used to understand the potential unintended consequences of the FDA’s e-cigarette authorization for market entry among young adults who do not use tobacco and inform policy decisions and communication strategies to reduce any such unintended consequences. With potentially more e-cigarettes to be authorized by the FDA through PMTAs in the near future, study results can also be used to help guide the authorization process with the goal of preventing e-cigarette use among young adults who do not use tobacco.

## METHODS

### Participant recruitment, eligibility, and screening

In-depth interviews (n=25) were conducted between February and July 2023 with young adults, aged 18–29 years, who do not use tobacco in Bethesda, Maryland, USA. Participants were recruited through various online channels, including social media sites (Facebook, LinkedIn, and Instagram) and Craigslist (a website that allows users to post classified advertisements). Participant eligibility criteria were: 1) aged 18–29 years; 2) reporting never experimenting with any tobacco products or never regularly using any tobacco products before and not having used any tobacco products in the past 30 days^15^; 3) being susceptible to using e-cigarettes (defined as the absence of a firm commitment to not use e-cigarettes)^15^; and 4) being proficient in reading and speaking English. Those who reported eye movement or alignment abnormalities were deemed ineligible due to an eye-tracking research task unrelated to this study. Eligible individuals were contacted by the study coordinator to schedule an in-person visit to the National Institutes of Health campus in Bethesda, Maryland, USA.

### Study procedure and in-depth interviews

**Figure 1:**
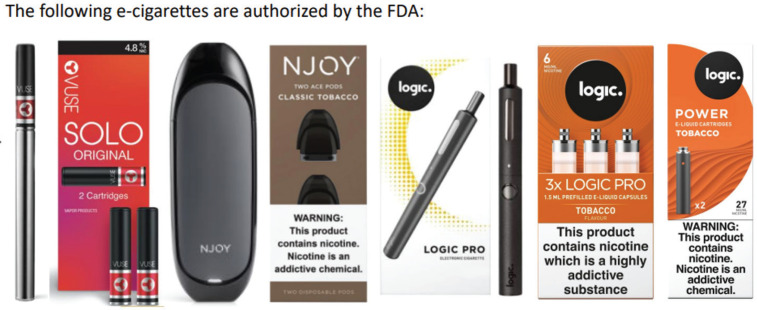
Stimuli of FDA-authorized e-cigarette products

Participants provided informed consent at the beginning of the study. Following this, they completed an online survey with questions related to their demographic information and tobacco use history. They then participated in one-on-one, in-depth interviews with a trained interviewer. The interviewer first showed participants an image of several e-cigarette products and their packages (all with tobacco or original flavors) that were authorized for market entry by the FDA at the time of the interview (Vuse Solo, NJOY ACE, Logic Pro, and Logic Power)^8^. The products and packages were directly obtained from e-cigarette brands’ official websites (Figure 1).

Using a semi-structured in-depth interview guide developed by the research team (Table 1), the interviewer asked questions to gather information on participants’ perceptions of and interest in using FDA-authorized e-cigarette products. The interviewer first asked participants to share what came to their mind when they first heard the phrase ‘FDA-authorized e-cigarettes’. They then asked the participants to make general comparisons between these FDA-authorized products and other e-cigarette products that they had seen in stores and advertisements. They also asked participants to discuss the harms of using FDA-authorized e-cigarettes compared to using other e-cigarette products. Participants were then asked about their interest in using FDA-authorized e-cigarettes in general and how they would compare their levels of interest in using authorized versus other e-cigarettes. Lastly, the interviewer inquired about participants’ interests in using these authorized e-cigarette products and if these products come in other flavors besides tobacco (e.g. fruit, candy, beverages). The interviewer also asked probing questions to gain a comprehensive understanding of participants’ thoughts and perceptions.

**Table 1:** In-depth interview questions used for current analysis

*Interview questions*
Q1. What came to your mind when you first heard ‘FDA-authorized e-cigarettes’? Q1.1 What does it mean if something is authorized by the FDA?
Q2. How would you compare these FDA-authorized e-cigarette products with other e-cigarettes that you’ve seen other people use, or that you’ve seen in advertisements and/or stores? Q2.1 How would you compare the harm of using these e-cigarette products that have been authorized by the FDA vs other e-cigarette products?
Q3. What do you think about using these e-cigarette products that have been authorized by the FDA? Q3.1 How would you describe your interest in using these e-cigarette products? Q3.2 How would you describe your interest in using these FDA-authorized e-cigarette products compared to other e-cigarette products?
Q4. These FDA-authorized e-cigarette products are currently only marketed with the original flavor. If they were also sold in other flavors such as menthol, mint, candy, coffee, or fruits, how would you describe your interest in using them?

The interviews were audio-recorded (about 30 minutes) with participants’ consent. After the interview, participants viewed health education materials about the harm of tobacco use and were compensated and dismissed. This study was reviewed by the Institutional Review Board of the National Institutes of Health and determined as exempted research.

### Interview data interpretation

This analysis includes interview data from 25 participants. Data saturation was reached around the 25th interview because no additional information emerged from the last few interviews^16^. Interview audio recordings were transcribed verbatim by a professional transcription company (Ubiqus.com). The transcripts were cross-checked against the recordings by the research team for accuracy. The transcripts were de-identified and imported to Dedoose^17^, a qualitative data management and analysis tool to prepare for the coding process. The research team used the Braun and Clarke^18^ 6-phase approach to thematic analysis, which was designed based on a constructionist epistemology, to guide the data coding and interpretation process.

Specifically, all members of the research team first familiarized themselves with the data by reading through the transcripts. Three trained coders from the research team first read through five transcripts multiple times to become familiar with the data and to develop an initial codebook consisting of thematic and content codes with definitions. The research team then met to discuss and confirm the completion of the codebook. Two coders then used the final codebook to double-code ten transcripts, reaching a satisfactory average coding agreement across codes (Krippendorff ’s alpha=0.76, percent agreement=89%). After a high coding agreement was established, the two coders separately coded the remaining 15 transcripts. Any coding disagreements were discussed and resolved by the third coder.

All members of the research team then independently identified patterns in the coded data and grouped the data into broad categories. The entire team then met to construct the themes based on the coded data and appropriately addressed the research questions. During this process, the team also named the themes, identified supporting participant quotes that accurately represented each theme, and presented them as a narrative to convey participants’ perceptions of and interests in using FDA-authorized e-cigarettes. All generated themes, their narratives, and their represented quotes were reviewed and agreed upon by the research team before final reporting.

## RESULTS

### Participant characteristics

Table 2 presents participant characteristics. For biological sex, over half of the participants were female (60%), and less than half were male (40%). Additionally, 16% were aged 18–21 years, 44% were 22–25 years, and 40% were 26–29 years. Self-identified race and ethnicity varied, as 32% of participants were White, 25% were Black, 32% were Asian, and 12% were Hispanic (any race) young adults. Over half of the participants had ever used an e-cigarette before (60%).

**Table 2:** Participant characteristics (N=25)

*Characteristics*	*Percent*
**Age** (years)	
18–21	16
22–25	44
26–29	40
**Biological sex**	
Male	40
Female	60
**Sexual orientation**	
Heterosexual	60
Non-heterosexual^a^	40
**Race/ethnicity**	
Hispanic	12
Non-Hispanic White	32
Non-Hispanic Black	24
Non-Hispanic Asian	32
Non-Hispanic Other^b^	0
**Education level**	
Lower than Bachelor’s degree	24
Bachelor’s degree	48
Higher than Bachelor’s degree	28
**Subjective financial status**	
Comfortable	56
Meet needs	44
Just meet needs	0
Do not meet needs	0
**E-cigarette ever use**	
Yes	60
No	40
**E-cigarette marketing exposure^c^**	
Never/rarely	20
Sometimes	52
Often	28

a Non-heterosexual includes homosexual, bisexual, other, and prefer not to say.

b Non-Hispanic Other includes non-Hispanic Native Hawaiian or Pacific Islander, American Indian or Alaskan Native, and multiple races.

c The cumulative frequencies of past 6-month e-cigarette marketing exposure through 8 marketing channels (e.g. websites and social media sites).

### Interview themes

Detailed interview themes are described below. Table 3 provides representative quotes for each theme labeled with participants’ biological sex and age.

**Table 3:** Themes and sample responses regarding reactions and perceptions toward FDA-authorized e-cigarettes

*Themes*	*Sample quotes*
**Low interest in using FDA-authorized e-cigarettes**	
Low interest in using FDA-authorized e-cigarettes currently on the market	*‘Yeah, I think that I feel like I can see their purpose as for someone who smoked cigarettes especially. I don’t think tobacco would be an appealing flavor. So I think that I would also not be interested on that standpoint.’* (Female, 22 years)
	*‘I’d probably be less interested. They seem to be all tobacco, nothing fruity with these, and the design choice is less stylish than the other one. Style of these look off-putting.’* (Male, 24 years)
	*‘Not really interested. I feel like even with the FDA authorization, it’s still an e-cigarette at the end of the day with the vaporized nicotine or some sort of tobacco derivative or whatever else may be in it. I don’t feel the desire to get a new addiction personally. So not really feeling too strongly about using it.’* (Female, 21 years)
Low interest in using hypothetical FDA-authorized e-cigarettes with other flavors	*‘If these were brighter and flavorful? I wouldn’t have an interest in them because I don’t want to try them, but I could see how they’re more appealing. If they did all that, it would be more appealing for people my age. But I wouldn’t try them.’* (Female, 22 years)
	*‘I would still say I have no interest in using them. I don’t think I’d want any of those artificial flavors, or I wouldn’t want them in, like, the vapor or an e-cigarette form. It still doesn’t interest me at all.’* (Male, 26 years)
	*‘I still wouldn’t be interested … the target audience would begin to lower in age because now you’re bringing candy and stuff like that. So it’s going to draw the attention of the younger crowds…’* (Male, 24 years)
	*‘I think that would decrease my interest in using them … in my college town, they banned the fruit flavor ones because they were so attractive to youth. And I do kind of look at it like it’s a trap that they’re trying to establish a pattern in their users so they will continually purchase this product.’* (Female, 25 years)
**FDA-authorized e-cigarettes are unappealing due to tobacco flavors and product design**	
Tobacco flavors reduce the appeal of FDA-authorized e-cigarettes	*‘This appears to be tobacco flavor and flavorless, or something like that, which seems pretty different to what I see people using … those all seem to be a lot more wildly flavored and pleasant.’* (Female, 28 years)
	*‘At the same time, I don’t think there’s as many flavors in these as I saw … so I don’t think they’re intended to taste or smell as good as the other ones so they’re less likely to be used by kids, I think.’* (Male, 21 years)
	*‘I mean, these seem they would appeal to someone who has smoked cigarettes before and is looking to not smoke cigarettes. I mean, they all say that they’re like tobacco flavored, they’re not like banana or something.’* (Female, 22 years)
	*‘These are all tobacco flavored. I feel like these are probably more for your cigarette smokers who start off as cigarette smokers and come over to e-cigs while the other ones were for non-cigarette smokers who still got into it.’* (Male, 24 years)
The product design of FDA-authorized e-cigarettes is not visually appealing	*‘Well, they’re certainly ugly. I think they look like batteries or pens or just like weird big blocks of things that I think – clearly, they’re missing out on the stylish element that other ones have, for sure.’* (Female, 18 years)
	*‘For starters, they’re a lot less colorful. They seem to be much more blatant; you know exactly what you’re buying. At least to me, this doesn’t seem as targeted toward kids or people just wandering into gas stations, like I feel like someone who’s buying these are probably more like veteran users.’* (Male, 22 years)
	*‘First of all, they seem a little toned down. I mean, in terms of the packaging, it’s in I guess a more limited color range. There is not as much yellow and blue and purple, which I think of as being kid-oriented colors.’* (Female, 28 years)
	*‘These kinda look dorky. They’re too silvery. They look like computer parts.’* (Male, 24 years)
**FDA authorization means the authorized products are safe to use**	
FDA authorization means quality control and a stamp of approval	*‘I think it would be easier to treat someone with e-cigarette use that has been approved by the FDA because, once again, all the compounds in there have been tested before so we know that all the compounds are and what their risks are.’* (Male, 21 years)
	*‘I guess having the authorization by the FDA means you can be assured that there is some sort of oversight to how these are developed. There’s some sort of rules in place, I guess, quality assurance maybe.’* (Female, 21 years)
	*‘I mean, they’re authorized by the FDA. I don’t really know exactly all that consists of their authorization. But I guess out of what is on the market for e-cigarettes broadly these are the safest option to use.’* (Female, 26 years)
	*‘I’m curious on why it’s authorized by the FDA. I’m curious what makes these different compared to others which makes the FDA one, authorize this, but two, doesn’t authorize other ones.’* (Male, 24 years)
	*‘But I’m still very skeptical and have a lot of questions because it doesn’t – I am a little confused about how they could authorize something because they still seem so harmful to health overall.’* (Female, 26 years)
FDA-authorized e-cigarettes are less harmful than other e-cigarettes	*‘Like I said before, it’s standardized. The assumption that there’s some quality assurance. There’s some rules behind how these are developed. There’s some restrictions. So I guess in that regard I’d assume it’s a little bit safer.’* (Female, 21 years)
	*‘I think that they might not have as many bad ingredients or they might have like, a safer cartridge where you’re not going to get hurt using it or it’s not going to blow up in your hand or anything like that.’* (Male, 26 years)
	*‘I would think the harm is slightly less because the flavors are not as pleasant to a non-smoker. One thing that I can imagine is if you’re vaping and it tastes like banana or fruit punch or something, you can go through a whole, I don’t know, pod, device, whatever the unit is, really quickly, because it’s pleasant.’* (Female, 28 years)

### Low interest in using FDA-authorized e-cigarettes

*Low interest in using FDA-authorized e-cigarettes and e-cigarettes in general*

Almost all participants expressed that they were not interested in trying FDA-authorized e-cigarettes mainly because they had no interest in using any type of e-cigarette in general. Some believe that all e-cigarettes, authorized or not, are meant to be used by those who smoke cigarettes. Some participants mentioned that they did not intend to use any e-cigarettes, including FDA-authorized e-cigarettes, because they contain nicotine and are harmful to health. Additional reasons for low use interest, specific to FDA-authorized e-cigarettes, included the unappealing tobacco flavors and product designs, which are discussed in greater detail in later themes. Although the majority shared no interest in using e-cigarette products in general, there were a few participants who expressed interest in trying FDA-authorized e-cigarettes because of perceived low harm and curiosity about what they taste like. The few who expressed more interest mentioned that they believed FDA-authorized e-cigarettes are less harmful to health compared to other e-cigarettes.

*Low interest in using hypothetical FDA-authorized e-cigarettes with other flavors*

When asked about their interest in using FDA-authorized e-cigarettes if they were to come with other flavors, such as fruit and candy, almost all participants mentioned they would not be interested in using the hypothetical flavored products. Many mentioned again that this is because they simply were not interested in using any e-cigarette products and that there are many harmful chemicals in e-cigarette flavors that may lead to negative health consequences. Many also mentioned that the hypothetical flavored products may be attractive to youth or individuals younger than them because of the strong appeal of flavors. This dampened the interest of these participants because they believed that they would not be a part of the intended target audience and also felt that adding flavors to the products is a trap by the tobacco industry to make young people addicted.

### FDA-authorized e-cigarettes are unappealing due to tobacco flavors and product design

*Tobacco flavors reduce the appeal of FDA-authorized e-cigarettes*

Although participants were not asked specific questions about product flavors in the images during the initial part of the interview, most brought up flavors when describing their first impressions of the FDA-authorized e-cigarettes. Many participants specifically said that the FDA-authorized e-cigarette products in the stimuli ‘seem bland in terms of flavor’. They further explained that these products differ from other e-cigarettes that they have seen people use, which come in ‘a variety of fun flavors’ and are ‘more wildly flavored and pleasant’ compared to the tobacco flavor of these FDA-authorized e-cigarettes. Many participants specifically said that flavors like banana, strawberry, mango, and other fruity flavors sound more appealing than tobacco flavors. Some also mentioned that e-cigarette products with other flavors are more socially acceptable than FDA-authorized e-cigarettes because ‘those don’t smell bad’. Additionally, some stated that the tobacco flavors made it clear to them that FDA-authorized e-cigarettes are primarily meant to target older people and those who smoke, not themselves. A few specified that FDA-authorized e-cigarettes may be favored among and beneficial for those who smoke cigarettes because of the familiar tobacco flavors.

*The product design of FDA-authorized e-cigarettes is not visually appealing*

Many participants also quickly noticed the differences between the design of the FDA-authorized products in the stimuli compared to other e-cigarettes that they saw in daily life. A majority specifically said that FDA-authorized e-cigarettes are not as ‘visually appealing’ or ‘stylish’ compared to other e-cigarettes due to their ‘limited color range’ and ‘bulky shapes’. Participants also said these FDA-authorized e-cigarettes look similar to ‘office supplies’, ‘computer parts’, ‘thumb drives or flash drives’, and ‘pens and pencils’. Some also mentioned that they did not find FDA-authorized e-cigarettes attractive because they are shaped like traditional cigarettes and believed these products are for those who smoke or older people.

### FDA authorization means the authorized products are safe to use

*FDA authorization means quality control and a stamp of approval*

Many participants interpreted FDA authorization to mean ‘product approval’. Specifically, participants used phrases such as ‘quality control’, ‘vetting process’, and ‘lab testing’ to describe what the FDA authorization process means to them. Some mentioned that from their understanding, the FDA regulates what chemicals and other ingredients go into the approved products and their effects on consumers’ health and safety. Many also expressed that they associated the FDA authorization process with approval and reassurance, especially because people are familiar with the FDA as a credible regulator of commercial products (i.e. drugs and medical devices). Some participants also mentioned that FDA authorization makes the products legal to use, and these authorized products are, therefore, not ‘sold illegally’. However, many participants also stated that they had never heard of the FDA authorizing e-cigarette products, expressed confusion and uncertainty about the authorization process, and mentioned they would like to know more about it. Additionally, a few shared that they were skeptical of the FDA or its authorization process and were surprised that e-cigarettes are being authorized because they believe that all tobacco and nicotine products are harmful to health.

*FDA-authorized e-cigarettes are less harmful than other (unauthorized) e-cigarettes*

When comparing the FDA-authorized e-cigarettes with other e-cigarettes that they saw in daily life (mostly unauthorized), most participants reported that they perceived FDA-authorized e-cigarettes to be less harmful to health. When asked why, participants often described that authorized products had undergone rigorous testing and review so the FDA could ‘track what’s in the products’ and make sure that there are no ‘harmful chemicals that have long-term health effects’. Participants also perceived FDA-authorized e-cigarettes to be less harmful because they do not have a variety of flavors or are ‘less addictive’. Some also explained that the lack of flavorings made the authorized products less appealing to young people, which may help prevent kids from using the products. Some expressed that FDA-authorized e-cigarettes are less harmful because they are authorized to help smokers quit.

## DISCUSSION

To our knowledge, this is one of the first studies to examine perceptions and use interests of FDA-authorized e-cigarettes among young adults who do not use tobacco. Our main finding suggests that the non-tobacco-using young adults in our study have low interest in using FDA-authorized e-cigarette products because they are not interested in using e-cigarettes or any tobacco product. Our results indicate that young adults may have little interest in using FDA-authorized e-cigarettes, especially due to their flavors and product design. Specifically, tobacco flavors and product designs of the authorized products were perceived as unappealing to the participants and contributed to their lack of interest in the products. This is unsurprising considering that sweet flavorings^19,20^ and colorful packaging^21,22^ of e-cigarette products are among the most important reasons for e-cigarette initiation and use among young people in the extant literature^23^. Therefore, based on the study results, the FDA is recommended to continue to evaluate the impact of flavorings and packages on e-cigarette product appeal through PMTAs and post-market surveillance to eventually reduce e-cigarette product use among young people who are new to tobacco products.

We found that many participants lacked knowledge about the FDA’s authorization process for market entry, expressed confusion, and raised questions about what FDA authorization means. Most participants believed that FDA authorization means product approval. Many participants also believed that FDA authorization means that the products were rigorously reviewed and do not contain chemical ingredients (e.g. flavorings, carcinogens) that pose a threat to human health. These findings suggest that FDA authorization may unintentionally miscommunicate approval for product use, which is inaccurate. FDA approval means that the FDA has decided that a product is safe and effective for use^24^ while authorization for market entry is granted to a product during urgent circumstances and if the benefits of its use outweigh the potential risks^25^. Therefore, public policy and communication efforts through effective messaging are greatly needed to correct this inaccurate belief because equating these two terms may contribute to misperceptions about the purpose of e-cigarette authorization and the health risks of FDA-authorized e-cigarettes.

Although some participants expressed confusion about the authorization for the market entry process, most claimed that they trusted the FDA in authorizing e-cigarette products and felt that the process was legitimate. Our findings, along with those from previous research, highlight that the public is accepting of the FDA as a tobacco regulator^26-28^. Relevantly, based on our results, the messages that e-cigarette products are for those who smoke cigarettes to quit may be an effective communication strategy to further reduce the interest in using the authorized products among young adults who do not use tobacco. Therefore, the FDA’s communications on those issues may help the public to further gain trust in the FDA’s authorization of e-cigarette products, reduce use interest among those who do not use tobacco, and promote complete product switching among those who smoke cigarettes.

Despite having trust in the FDA and the authorization for the market entry process, many participants had low interest in using FDA-authorized e-cigarettes because of their firm beliefs about the harms of e-cigarette products, in general. Thus, participants’ high harm perceptions towards e-cigarette products may directly serve to minimize their interest in using e-cigarette products under various hypothetical regulatory contexts. Specifically, although many participants thought that authorized e-cigarette products are less harmful than other (unauthorized) e-cigarette products, these reduced harm perceptions did not transfer to higher interest in using the authorized products. A similar lack of interest was found when asking participants to discuss their interest in using hypothetical authorized e-cigarette products that come with appealing flavors such as fruit and candy. Participants communicated their firm belief that e-cigarettes with flavoring chemicals are particularly harmful to health, even if they are authorized by the FDA. Additionally, we speculate that these harmful perceptions about e-cigarettes in general might be partially attributed to the widespread dissemination of campaigns (e.g. Truth Campaign and Real Cost Campaign) highlighting the harm and addictive potential of e-cigarette use among young people^29,30^. Further dissemination of these public education messages may reinforce the harmful perceptions of using e-cigarettes among young people who do not use tobacco.

### Limitations

The following limitations should be considered when interpreting the study results. First, we recruited a diverse rather than representative sample, although sample representation is not a goal of qualitative work^31^. In particular, we recruited our study participants from Maryland, a state with the lowest rate of e-cigarette use in the US^32^. Our study’s sample and their general lack of interest in using e-cigarettes generally may not be reflective of e-cigarette use interests among US young adults overall. It is also possible that participants’ responses about having no interest in using the products were influenced by social desirability bias^33^ as this was a face-to-face interview on the campus of the National Institutes of Health. Participants may have presented responses that they perceived to be viewed more favorably by the interviewer rather than their actual opinions. Second, the stimuli used in the study only included packages and product images and the fact that those e-cigarettes are authorized by the FDA. Participants’ responses may differ if they received more information that FDA authorization means the products are permitted to be marketed and sold in the US but not approved for use. This lack of mention may have confused the participants between ‘authorization’ and ‘approval’. However, in the real world, it is very likely that young adults will not be given the full information, especially when FDA authorization appears in the news or in commercial marketing materials. Third, participants’ reactions might have been different if the product images were to appear in commercial advertisements with other appealing marketing features, such as human models, nicotine warnings, and price promotions^15,34^. Lastly, the study did not include a youth sample whose perceptions of FDA authorization of e-cigarettes are also important to assess. Youth may show differential responses to the visual stimuli and the concept of FDA authorization compared to young adult participants.

## CONCLUSIONS

Currently, FDA-authorized e-cigarette products may not instigate increased e-cigarette use interests mainly because young adults who do not use tobacco have no interest in using any e-cigarette product in general. Additionally, participants may especially not be interested in using FDA-authorized e-cigarettes because of their unattractive tobacco flavors and designs. Therefore, the FDA is recommended to continue to evaluate the impact of flavorings and product design on e-cigarette product appeal through PMTAs and post-market surveillance to ensure that product authorization for market entry does not instigate use among young people. Additionally, we found that FDA authorization may unintentionally communicate product approval and safety to young adults in our study, but this did not change participants’ interest levels in using any type of e-cigarette product. The FDA should consider public education and communication about the purpose of FDA authorization, including the potential benefits of switching to these products for those who smoke and the potential harms of using e-cigarettes for those who do not use tobacco. More research is also greatly needed to understand whether and how perceptions related to the FDA authorization of e-cigarette products can promote complete product switching among those who smoke cigarettes.

## Data Availability

The data supporting this research are available from the authors on reasonable request.

## References

[1] Patrick ME, Schulenberg JE, Miech RA, Johnston LD, O’ Malley PM, Bachman JG. Monitoring the Future Panel Study Annual Report: National Data on Substance Use among Adults Ages 19 to 60, 1976 - 2021. The University of Michigan Institute for Social Research; 2022

[2] National Center for Chronic Disease Prevention and Health Promotion. Health Effects of E-Cigarette Use Among U.S. Youth and Young Adults. In: E-Cigarette Use Among Youth and Young Adults: A Report of the Surgeon General [Internet]. Centers for Disease Control and Prevention (U.S.); 2016. Accessed September 21, 2023. https://www.ncbi.nlm.nih.gov/books/NBK538688/

[3] McMillen R, Klein JD, Wilson K, Winickoff JP, Tanski S. E-Cigarette Use and Future Cigarette Initiation Among Never Smokers and Relapse Among Former Smokers in the PATH Study. Public Health Rep. 2019;134(5):528-536. doi:10.1177/0033354919864369 31419184 PMC6852065

[4] The U.S. Food and Drug Administration. FDA Permits Marketing of E-Cigarette Products, Marking First Authorization of Its Kind by the Agency. FDA. Published October 18, 2021. Accessed June 12, 2024. https://www.fda.gov/news-events/press-announcements/fda-permits-marketing-e-cigarette-products-marking-first-authorization-its-kind-agency

[5] The U.S. Food and Drug Administration. Premarket Tobacco Product Applications. FDA. August 23, 2022. Accessed March 23, 2023. https://www.fda.gov/tobacco-products/market-and-distribute-tobacco-product/premarket-tobacco-product-applications

[6] The U.S. Food and Drug Administration. Premarket Tobacco Product Applications. FDA. March 27, 2024. Accessed June 6, 2024. https://www.fda.gov/tobacco-products/market-and-distribute-tobacco-product/premarket-tobacco-product-applications

[7] The U.S. Food and Drug Administration. Section 910 of the Federal Food, Drug, and Cosmetic Act - Application for Review of Certain Tobacco Products. FDA. December 8, 2022. Accessed September 21, 2023. https://www.fda.gov/tobacco-products/rules-regulations-and-guidance/section-910-federal-food-drug-and-cosmetic-act-application-review-certain-tobacco-products

[8] The U.S. Food and Drug Administration. Premarket Tobacco Product Marketing Granted Orders. FDA. June 14, 2023. Accessed July 7, 2023. https://www.fda.gov/tobacco-products/premarket-tobacco-product-applications/premarket-tobacco-product-marketing-granted-orders

[9] The U.S. Food and Drug Administration C for T. The Tobacco Product Applications: Metrics & Reporting. FDA. February 15, 2024. Accessed February 26, 2024. https://www.fda.gov/tobacco-products/market-and-distribute-tobacco-product/tobacco-product-applications-metrics-reporting

[10] Glantz S, Lempert LK. Vuse Solo e-cigarettes do not provide net benefits to public health: a scientific analysis of FDA’s marketing authorization. Tob Control. 2023:tobaccocontrol-2022-057296. doi:10.1136/tc-2022-057296 PMC10409877 36764683

[11] Meshnick AB, Faricy LE, Lushniak BD. Analysis of FDA’s Vuse market authorization: limitations and opportunities. Tob Control. 2023;tobaccocontrol-2022-057540. doi:10.1136/tc-2022-057540 36764685

[12] Weiger C, Chen-Sankey J, Jeong M, Delnevo C, Wackowski O. Awareness and beliefs about FDA e-cigarette regulation in the premarket application review era. Addict Behav. 2023;144:107748. doi:10.1016/j.addbeh.2023.107748 37182238 PMC10330513

[13] Lee S, Xie Z, Xu E, Shao Y, Ossip DJ, Li D. Public perceptions of the FDA’s marketing authorization of Vuse on Twitter/X. Front Public Health. 2023;11:1280658. doi:10.3389/fpubh.2023.1280658 38026290 PMC10654997

[14] Wackowski OA, Jeong M, Gratale SK, et al. The impact of exposure to FDA e-cigarette authorization messages on product perceptions and interest - an experiment with adults who smoke and youth. Nicotine Tob Res. doi:10.1093/ntr/ntae141 PMC13032063 38836598

[15] Chen-Sankey J, Jeong M, Wackowski OA, et al. Noticing people, discounts and non-tobacco flavours in e-cigarette ads may increase e-cigarette product appeal among non-tobacco-using young adults. Tob Control. 2023;33(1):30-37. doi:10.1136/tobaccocontrol-2022-057269 35672144 PMC9726993

[16] How Many Interviews Are Enough?: An Experiment with Data Saturation and Variability - Greg Guest, Arwen Bunce, Laura Johnson, 2006. Accessed January 17, 2024. https://journals.sagepub.com/doi/abs/10.1177/1525822x05279903

[17] Great Research Made Easy. Dedoose. Accessed March 24, 2023. https://www.dedoose.com/

[18] Braun V, Clarke V. Thematic analysis. In: Cooper H, Camic PM, Long DL, Panter AT, Rindskopf D, Sher KJ, eds. APA Handbook of Research Methods in Psychology, Vol 2: Research Designs: Quantitative, Qualitative, Neuropsychological, and Biological. American Psychological Association; 2012:57-71. doi:10.1037/13620-004

[19] Landry RL, Groom AL, Vu TT, et al. The role of flavors in vaping initiation and satisfaction among U.S. adults. Addict Behav. 2019;99:106077. doi:10.1016/j.addbeh.2019.106077 31437770 PMC6903386

[20] Gupta PS, Kalagher KM. Where There Is (No) Smoke, There Is Still Fire: a Review of Trends, Reasons for Use, Preferences and Harm Perceptions of Adolescent and Young Adult Electronic Cigarette Use. Curr Pediatr Rep. 2021;9(3):47-51. doi:10.1007/s40124-021-00240-1 33996271 PMC8107807

[21] Taylor E, Arnott D, Cheeseman H, et al. Association of Fully Branded and Standardized e-Cigarette Packaging With Interest in Trying Products Among Youths and Adults in Great Britain. JAMA Netw Open. 2023;6(3):e231799. doi:10.1001/jamanetworkopen.2023.1799 36917111 PMC10015302

[22] Laverty AA, Vardavas CI, Filippidis FT. Design and marketing features influencing choice of e-cigarettes and tobacco in the EU. Eur J Public Health. 2016;26(5):838-841. doi:10.1093/eurpub/ckw109 27471217 PMC5054276

[23] Patel D, Davis KC, Cox S, et al. Reasons for current E-cigarette use among U.S. adults. Prev Med. 2016;93:14-20. doi:10.1016/j.ypmed.2016.09.011 27612572 PMC5316292

[24] The U.S. Food and Drug Administration O of the. About FDA Product Approval. FDA. November 3, 2018. Accessed February 27, 2024. https://www.fda.gov/news-events/approvals-fda-regulated-products/about-fda-product-approval

[25] The U.S. Food and Drug Administration. Emergency Use Authorization. FDA. February 22, 2024. Accessed February 27, 2024. https://www.fda.gov/emergency-preparedness-and-response/mcm-legal-regulatory-and-policy-framework/emergency-use-authorization

[26] Osman A, Kowitt S, Sheeran P, Jarman K, Ranney L, Goldstein A. Information to Improve Public Perceptions of the Food and Drug Administration (FDA’s) Tobacco Regulatory Role. IJERPH. 2018;15(4):753. doi:10.3390/ijerph15040753 29661991 PMC5923795

[27] Schmidt AM, Jarman KL, Ranney LM, et al. Public Knowledge and Credibility Perceptions of the FDA as a Tobacco Regulator. Nicotine & Tobacco Research. 2018;20(11):1310-1316 29059369 10.1093/ntr/ntx215 PMC6154977

[28] Yingst J, Veldheer S, Foulds J. Knowledge and Perceptions of FDA Tobacco Regulation among U.S. Adults in 2015. Journal of Regulatory Science. 2018:15-19. doi:10.21423/JRS-V06N01P015

[29] The U.S. Food and Drug Administration. Looking Back, Looking Ahead: FDA’s Progress on Tobacco Product Regulation in 2022. FDA. February 24, 2023. Accessed January 25, 2024. https://www.fda.gov/tobacco-products/ctp-newsroom/looking-back-looking-ahead-fdas-progress-tobacco-product-regulation-2022

[30] The U.S. Food and Drug Administration. E-Cigarettes, Vapes, and other Electronic Nicotine Delivery Systems (ENDS). FDA. December 18, 2023. Accessed January 25, 2024. https://www.fda.gov/tobacco-products/products-ingredients-components/e-cigarettes-vapes-and-other-electronic-nicotine-delivery-systems-ends

[31] Gentles S, Charles C, Ploeg J, McKibbon KA. Sampling in Qualitative Research: Insights from an Overview of the Methods Literature. TQR. November 7, 2015. doi:10.46743/2160-3715/2015.2373 PMC5059917 27729071

[32] American Lung Association. Rates By State. February 2, 2023. Accessed January 22, 2024. https://www.lung.org/research/trends-in-lung-disease/tobacco-trends-brief/rates-by-state

[33] Bergen N, Labonté R. ‘Everything Is Perfect, and We Have No Problems’: Detecting and Limiting Social Desirability Bias in Qualitative Research. Qual Health Res. 2020;30(5):783-792. doi:10.1177/1049732319889354 31830860

[34] La Capria K, Uriarte C, Elhabashy M, et al. Exploring the Influence of E-cigarette Ad Features on Perceived Product Appeal and Use Interest Among Young Adults of Varying Tobacco-Use Behaviors. Nicotine Tob Res. 2023:ntad150. doi:10.1093/ntr/ntad150 PMC10882433 37594249

